# Identifying incident cancer cases in dispensing claims: A validation study using Australia’s Repatriation Pharmaceutical Benefits Scheme (PBS) data

**DOI:** 10.23889/ijpds.v5i1.1152

**Published:** 2019-03-19

**Authors:** B Daniels, HE Tervonen, S-A Pearson

**Affiliations:** 1 Medicines Policy Research Unit, Centre for Big Data Research in Health, UNSW, Sydney, Australia

## Abstract

**Introduction:**

Dispensing claims are used commonly as proxy measures in pharmacoepidemiological studies; however, their validity is often untested.

**Objectives:**

To assess the performance of a proxy for identifying cancer cases based on the dispensing of anticancer medicines and estimate the misclassification of cancer status and potential for bias researchers may encounter when using this proxy.

**Methods:**

We conducted our validation study using Department of Veterans’ Affairs (DVA) client data linked with the New South Wales (NSW) Cancer Registry and Repatriation Pharmaceutical Benefits Scheme data. We included DVA clients aged ≥65 years residing in NSW between July 2004 and December 2012. We matched clients with a cancer diagnosis to clients without a diagnosis based on demographic characteristics and available observation time. We used dispensing claims for anticancer medicines dispensed between July 2004 and December 2013 as a proxy to identify clients with cancer and calculated sensitivity, specificity, positive predictive values and negative predictive values compared with cancer registrations (gold standard), overall and by cancer site. We illustrated misclassification by the proxy in a cohort of people initiating opioid therapy. Using the proxy, we excluded people with cancer from the cohort, in an attempt to delineate people potentially using opioids for cancer rather than chronic non-cancer pain.

**Results:**

We identified 15,679 new cancer diagnoses in 14,112 DVA clients from the cancer registry and 62,663 clients without a diagnosis. Sensitivity of the proxy based on dispensing claims was 30% for all cancers and around 20% for specific cancers (range: 10-67%). Specificity was above 90% for all cancers. The dispensing proxy correctly identified 26% of people with a cancer diagnosis who initiated opioid therapy and failed to identify 74% those with a cancer diagnosis; the proxy was most robust for clients with breast cancer where 61% were correctly identified by proxy.

**Conclusions:**

Using dispensing of anticancer medicines to identify people with a cancer diagnosis performed poorly. Excluding patients with evidence of anticancer medicine use from cohort studies may result removal of a disproportionate number of women with breast cancer. Researchers excluding or otherwise using anticancer medicine dispensing to identify people with cancer in pharmacoepidemiological studies should acknowledge the potential biases introduced to their findings.

**Keywords:**

cancer, diagnosis, proxy, dispensing records, validation study

## Introduction

Pharmacoepidemiological studies utilising routinely-collected medicines data are essential for examining the use and impact of medicines in real-world clinical practice [1]. However, the utility of dispensing claims in particular remains relatively limited unless they are linked with other collections such as hospitalisation data, disease and death registries. Researchers undertaking studies solely in dispensing data rely frequently on evidence of specific medicine dispensing as a proxy measure for comorbidity, disease indications, and outcomes [2, 3]. In Australia, pharmacoepidemiological studies are often undertaken in Pharmaceutical Benefits Scheme (PBS) data [4] that are not linked to other data sources and the use of proxies based on dispensing claims is widespread [5]. A range of studies have examined the validity of different proxies in PBS data with varying conclusions regarding their robustness [3, 6-10].

Proxy measures are especially pertinent for pharmacoepidemiological studies attempting to differentiate indications for medicine use. There is often a need to identify people with cancer — whether to specifically examine outcomes in a group of people with cancer or to exclude or otherwise account for people who have a cancer history or not. Cancer registries are the ‘gold standard’ for identifying incident cancer cases in specific geographic areas [11, 12]. Registries receive information from multiple sources and trained medical coders process the information following international conventions. However, the process of producing complete and accurate cancer incidence data often means that there is a significant time lag in availability (2.5-3 years in Australia)[13], even if it can be linked with other collections [14]. If registry data are not available or sufficiently up-to-date, researchers seeking to identify people with cancer must rely on proxies to do so.

Several Australian studies have used the dispensing of anticancer medicines as a proxy for incident cancer [15, 16], notably studies investigating patterns of opioid use [17-19]. While the use of this proxy is common and intuitively supported by the specific indications of anticancer medicines—few, if any, of these agents are indicated for conditions other than cancer— its validity has not been investigated comprehensively nor are the implications of applying such a proxy well understood. Previous Australian studies have shown that using the dispensing of anticancer medicines as a proxy for cancer diagnosis results in low sensitivities and positive predictive values for lung, colorectal, and breast cancers [20, 21]. Considering the wide range of cancer treatments and treatment modalities, it is likely that ascertainment of cancer cases using medicine dispensing data also varies by cancer site and including or excluding people based on dispensing proxies may create biases.

A comprehensive evaluation of this proxy across all reportable cancer sites, and the implications of using such a proxy in medicines research in Australia are currently lacking. Therefore, in this study we used gold-standard cancer registry data linked with Repatriation PBS (RPBS) dispensing records to achieve the following:

Aim 1) To assess the performance of a proxy for identifying cancer cases based on the dispensing of anticancer medicines. We calculated the sensitivity, specificity, positive predictive value (PPV), and negative predictive value (NPV) for the proxy across all cancers (any cancer diagnosis) as well as by specific cancer sites and

Aim 2) To quantify the amount of cancer status misclassification and potential for bias that researchers may encounter when using this proxy. As a motivating example to understand the implications of the proxy in practice, we identified a cohort of people initiating opioid therapy and determined cancer status in an attempt to delineate people potentially using opioids for cancer rather than chronic non-cancer pain. We estimated the misclassification of cancer based on the proxy according to look-back periods of different lengths. 

## Methods

### Study setting and data source

Australia maintains a universal healthcare system, entitling all Australian citizens and permanent residents to subsidised prescription medicines, community health services, and hospital care. In addition, the Australian Government Department of Veterans’ Affairs (DVA) funds the healthcare and pharmaceutical items for eligible veterans, war widows/widowers and their dependents. DVA clients may be eligible for services that are not funded for the general population.

Information about the DVA, its clients, and the datasets comprising our data holding have been described in detail in our research protocol paper [22]. Briefly, we used the DVA client database linked with the New South Wales Cancer Registry (NSWCR) and the Repatriation Schedule of Pharmaceutical Benefits (RPBS) dispensing data. The RPBS provides access to all pharmaceutical items available to the general community under the Pharmaceutical Benefits Scheme (PBS), as well as additional medicines available only to veterans [23]. The PBS is a national program that provides subsidised access to approved medicines for all Australians. The DVA client database contains information for all Australians eligible for DVA-funded benefits, including residential history. The NSWCR includes information about all primary malignant cancer cases diagnosed in NSW, the most populous state of Australia. The NSWCR receives information from pathology laboratories, public and private hospitals, radiotherapy and medical oncology departments, aged care facilities, day procedure centres and the NSW Registry of Births, Deaths and Marriages [24]. The time period observed in the data is July 2004 through December 2013. The Centre for Health Record Linkage (CHeReL) used probabilistic linkage to perform the linkage [22].

### Study cohort

We restricted our analyses to those clients receiving Repatriation Gold Card benefits as these clients are able to access prescribed medicines with a reduced co-payment amount, meaning all of their dispensed medicines are captured in our dispensing data from the time their Gold Card benefits began [22]. As the DVA client population is predominantly over 65 years of age, we included all DVA clients aged ≥65 years at the time their gold card benefits began who resided in NSW between July 2004 and December 2012 [22]. We excluded clients leaving NSW at any point after starting Gold Card benefits, as we were only able to capture cancer diagnoses for patients residing in NSW.

### Ascertainment of cancer and anticancer medicine dispensing

We determined all primary invasive cancers diagnosed in our cohort from the NSWCR data, using the International Classification of Diseases, Australian Modification (ICD-10-AM) codes to identify cancer sites. These diagnoses data from the NSWCR comprised the gold standard for evaluating our anticancer medicines dispensing proxy. We excluded clients who were diagnosed at death or died within one month of diagnosis because they may not have had sufficient time or been robust enough to receive anticancer treatment or have been admitted to public hospitals, in which case their anticancer medicines use would not be captured in the dispensing data.

For our dispensing proxy, we used evidence of at least one anticancer medicine dispensing as indicating a cancer diagnosis. We defined anticancer medicines as those with Anatomic Therapeutic Chemical Classification System (ATC) codes beginning with L01, L02, L03 and L04. As medicines falling under the L03 and L04 classification have non-cancer indications we performed a separate analysis focusing on just medicines whose ATC codes begin with L01 and L02, which are only indicated for the treatment of cancer. In this paper, we present the results from this analysis focused on L01 and L02 medicines, with the results from the analysis incorporating L01, L02, L03, and L04 medicines presented in the supplement.

### Statistical analyses

We used the following variables in our analyses: sex, age (at time of Gold Card start date), year of birth, month and year of diagnosis, and date of death. We calculated the sensitivity, specificity, PPV and NPV for our proxy against the gold standard NSWCR data overall and by cancer site. We defined: sensitivity as the probability of being identified as cancer case by dispensing proxy when cancer was present according to the NSWCR data; specificity as the probability of being not identified as cancer case when cancer is not present; PPV as the probability of having cancer when identified as cancer case by the proxy; and NPV as the probability of not having cancer when not identified as cancer case by the dispensing proxy.

**Figure 1: Diagram illustrating the matching of beneficiaries with a cancer diagnosis to those without a cancer diagnosis:**
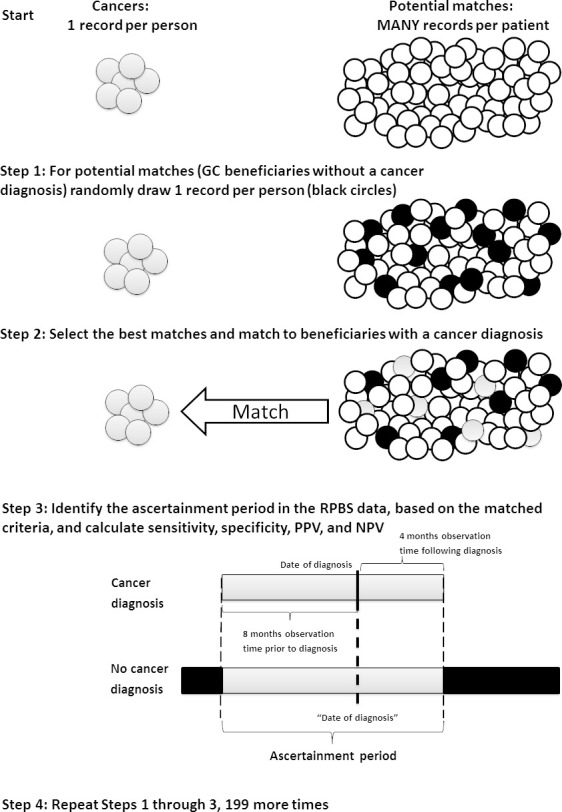
The period of time in the data selected to ascertain the presence or absence of an anticancer medicine dispensing; and the iterative nature of the process. GC = Gold Card; RPBS = Repatriation Pharmaceutical Benefits Scheme; PPV = positive predictive values; NPV = negative predictive value.

To control for demographic characteristics, changes in treatment patterns over time, and different observation periods in the dispensing data for different clients, we matched clients with and without a cancer diagnosis (Figure 1). Clients with cancer were matched to those without cancer diagnosis, one-to-one, exactly on sex and month/year of diagnosis; and then matched using nearest-neighbour propensity score matching based on their year of birth, available observation time prior to diagnosis to a maximum of 12 months (i.e., from Gold Card start date or 12 months prior to diagnosis, whichever was shorter), and available follow-up time after diagnosis to a maximum of 12 months (i.e., from diagnosis until death or 12 months following diagnosis, whichever was shorter). Ascertainment of cancer diagnosis based on anticancer medicine dispensing was restricted to a maximum of this 24-month period.

For matching purposes, we treated each month that a client without cancer diagnosis was alive as a potential “month of diagnosis”. For instance, if a non-cancer, female client became a Gold Card holder in January 2005 and died in January 2010, they may have been matched to a female client who was diagnosed with cancer in September 2005 and died four months later. In this scenario, the non-cancer client has a “month of diagnosis” for matching purposes of September 2005, eight months of available observation time for dispensing records prior to “diagnosis”, and four months following “diagnosis”—corresponding to the available data for the client with a cancer diagnosis (Figure 1).

As non-cancer clients could have multiple ascertainment periods, we iteratively extracted 200 random samples of non-cancer clients, created 200 one-to-one matches with cancer clients, and calculated estimates of sensitivity, specificity, PPV, and NPV for each sample (Figure 1). We averaged the resulting 200 estimates of sensitivity, specificity, PPV, and NPV to arrive at our reported results [25]. We derived these bootstrap samples and calculated these measures for all cancers together and by specific cancer site.

### Implications for practice

We further explored the potential for bias associated with using the proxy by constructing a motivating example study of opioids utilisation. For this example, we wanted to select a cohort of Gold Card-holding, NSW residents ≥65 without a cancer diagnosis who initiate opioid treatment. We applied the anticancer medicine proxy to several look-back periods (3, 6, and 12months) to identify clients with a cancer diagnosis prior to their incident opioid dispensing. A client was considered to be initiating opioid therapy at their first opioid dispensing following a look-back period (3, 6, and 12 months, corresponding to the look-back period for the proxy) with no opioid dispensing. To understand the impact of using the proxy in this plausible research scenario, we then quantified the proportion of misclassified clients by each look-back period and cancer site and explored the characteristics of clients with cancer as identified in the NSWCR and those not identified as having a cancer diagnosis by the dispensing proxy. We performed two analyses of misclassification—one that included only those cancers diagnosed within the look-back period (so, cancers diagnosed up to 3, 6, and 12 months prior to incident opioid dispensing) and another that included any cancer diagnoses prior to incident opioid dispensing. In the remainder of the paper, we focus on the results of 12-month look-back period.

Analyses were conducted using SAS version 9.4 (SAS Institute, Cary, NC) and R version 3.5.1.

### Ethics approval, data access, and consent to participate.

Our study was approved by the NSW Population and Health Services Research Ethics Committee (Approval Number: 2013/11/494) and The Departments of Defence and Veterans’ Affairs Ethics Committee (Approval number: E013/015). The Ethics Committees granted a waiver of the usual requirement for individual consent to the use of their health information in a research project, in line with the National Statement on Ethical Conduct in Human Research (2007) (Chapter 2.3) and the Guidelines approved under Section 95/95A of the Privacy Act 1988.

## Results

We identified 76,775 DVA clients who resided in NSW continually from the start of Gold Card benefits during the study period. Of these clients, 14,112 (18%) had at least one incident cancer diagnosis (15,679 diagnoses in total) in the NSWCR; 62,663 (82%) did not have a cancer diagnosis. Urogenital cancers were the most frequently occurring diagnosis, followed by skin cancers and colorectal cancers. The mean age of clients with a cancer diagnosis was 83.7 (SD: 5.7) years and 84.9 (SD: 6.1) years in those without a cancer diagnosis. The majority of our cohort was male (65%). Clients with a cancer diagnosis had an average of 11.8 (SD: 1.0) months of observation time prior to diagnosis and 9.5 (4.0) months of observation time following diagnosis; while clients without a cancer diagnosis selected for matching had an average of 11.0 (SD: 2.8) months and 9.0 (4.4) months of observation time prior to and following the month used as “diagnosis” for matching, respectively (Table 1).

**Table 1: Characteristics of DVA clients with cancer diagnosis and matched clients without a cancer diagnosis:** © Commonwealth of Australia 2019. * Standard deviation † Month of diagnosis for clients without a cancer diagnosis was the month selected for matching purposes.

	Clients with a cancer diagnosis	Matched clients without a cancer diagnosis
N	14,112	62,663
Number of diagnoses	15,679	-
Urogenital	3,305	-
Skin	3,268	-
Colorectal	2,241	-
Lymphomas	1,683	-
Respiratory	1,644	-
Upper gastrointestinal	1,236	-
Breast	899	-
Cancer of unknown primary	471	-
Gynaecological	343	-
Head & neck	316	-
Neurological	120	-
Endocrine	61	-
Bone	58	-
Eye	34	-
Mean age (SD*)	83.7 (5.7)	84.9 (6.1)
% Female	35.4%	35.4%
Mean (SD) observation time in months prior to diagnosis†	11.8 (1.0)	11.0 (2.8)
Mean (SD) observation time in months following diagnosis†	9.5 (4.0)	9.0 (4.4)

The sensitivity for detecting any cancer diagnosis (all sites) using the proxy was 29.1% and was similarly low for most cancer sites (Table 2). Specificity of the proxy for all cancers was 93.6% and above 90% for each cancer site. The PPV of the proxy was 83.4% for all cancers and ranged from 72.4% to 93.8% for specific cancer sites; while the NPV was 54.4% for all cancers and ranged from 42.2% to 71.7% for the different cancer sites. Breast cancers were the only cancers for which sensitivity was above 50% (67.1%) and the NPV higher than 60% (71.7%).

**Table 2: Sensitivity, specificity, positive predictive value (PPV), and negative predictive value (NPV) estimates for the anticancer medicines dispensing proxy:** © Commonwealth of Australia 2019. * Where an individual had multiple cancers with the same date of diagnosis, just one was included in the all-cancers analysis. † Positive predictive value ‡ Negative predictive value

	Number of cancer cases and matched non-cancer cases	Sensitivity	Specificity	PPV†	NPV‡
All cancers*	15,513	29.1%	93.6%	83.4%	54.5%
Urogenital	3,305	44.0%	93.2%	88.6%	58.4%
Skin	3,268	18.2%	93.3%	76.6%	48.6%
Colorectal	2,241	22.6%	93.8%	82.7%	48.2%
Lymphomas	1,683	39.8%	93.8%	89.4%	54.4%
Respiratory	1,644	23.1%	93.8%	82.5%	49.2%
Upper gastrointestinal	1,236	18.3%	94.2%	79.7%	48.0%
Breast	899	67.1%	94.9%	93.8%	71.7%
Cancer of unknown primary	471	16.7%	94.4%	79.3%	47.1%
Gynaecological	343	23.4%	94.8%	84.9%	50.0%
Head & neck	316	19.4%	93.6%	81.0%	45.6%
Neurological	120	21.6%	93.9%	80.9%	51.0%
Endocrine	61	9.9%	94.3%	72.4%	42.2%
Bone	58	19.2%	94.8%	84.9%	44.8%
Eye	34	27.5%	94.1%	85.7%	51.8%

In our motivating example, we identified 31,795 clients initiating opioid therapy with a 12-month look-back period, 52,058 with a 6-month look-back period, and 71,903 with a 3-month look-back period. We used the same look-back periods to identify cancer cases using dispensing records and found the ascertainment of true cancer cases decreased with shorter look-back periods (Table 3). Of the 31,795 clients initiating opioid treatment with a 12-month look-back period, 1,395 (4%) had a cancer diagnosis in the NSWCR during the 12 months preceding opioid initiation and 5,453 (17%) had a cancer diagnosis at any point^1^ prior to initiating opioids (Table 3). The cancer proxy based on dispensing claims correctly identified 359 (26%) of those with a diagnosis in the preceding 12 months and failed to identify 1,036 (74%) clients. Among clients with a cancer diagnosis at any time prior to initiating opioids, 25% were correctly identified by using the proxy with a 12-month look-back period (Table 3).

**Table 3: Classification of people initiating opioids with and without a cancer diagnoses by lookback period:** © Commonwealth of Australia 2019.

	Opioid initiators with cancer, as classified by proxy	Opioid initiators without cancer, as classified by proxy	Clients with cancer diagnosis, correctly classified by proxy (%)	Clients with cancer diagnosis, incorrectly classified by proxy (%)
12-month lookback:
Cancers diagnosed within 365 days prior to incident opioid dispensing	424	31,371	359 (26)	1,036 (74)
Cancers diagnosed at any point prior to incident opioid dispensing	2,817	28,989	1,343 (25)	4,110 (75)
6-month lookback:
Cancers diagnosed within 180 days prior to incident opioid dispensing	337	51,721	270 (19)	1,121 (81)
Cancers diagnosed at any point prior to incident opioid dispensing	3,410	48,648	1,720 (21)	6,591 (79)
3-month lookback:
Cancers diagnosed within 90 days prior to incident opioid dispensing	195	71,708	154 (14)	961 (86)
Cancers diagnosed at any point prior to incident opioid dispensing	3,456	68,447	1,799 (17)	8,955 (83)

The characteristics of clients correctly identified as having a cancer diagnosis and those not identified were similar, though misclassified cancer cases were slightly older than those correctly identified as cancer cases (median age of 86 compared to 84; Table 4). Breast cancer diagnoses comprised 5% of all diagnoses in people initiating opioid therapy compared with 11% of all diagnoses in opioid initiators with cancer classified by proxy. Breast cancer was the only site of diagnosis for which the proxy correctly classified more clients initiating opioid therapy than it misclassified—61% of breast cancers correctly classified compared with 39% misclassified as no cancer diagnosis.

**Table 4: Cohort characteristics at opioid initiation according to anticancer medicine dispensing proxy (L01 and L02) and true cancer status (12-month look back period):** © Commonwealth of Australia 2019. * Percentages for site of primary cancer by correct or incorrect proxy identification are out of the total number of each cancer site. For instance, there were 210 colorectal cancer diagnoses in total, 39 (19%) of which were correctly identified and 171 (81%) which were not. The other cells in the table for age and sex use the column totals, specified in the “Opioid initiators” row, as the denominator for all percentages. † Cell counts less than 5 cannot be reported due to ethical restrictions.

12-month lookback	Opioid initiators with cancer, as classified by proxy	Opioid initiators without cancer, as classified by proxy	Clients with cancer diagnosis, correctly classified by proxy (%)	Clients with cancer diagnosis, incorrectly classified by proxy (%)
Opioid initiators	424	31,380	359	1,036
Median (IQR) age	84 (81 - 88)	85 (79 - 88)	84 (81 - 88)	86 (82 - 89)
N (%)
65 – 74*	35 (8)	3,154 (10)	30 (8)	81 (8)
75 – 84	182 (43)	10,200 (33)	155 (43)	316 (30)
85+	209 (49)	18,026 (57)	176 (49)	646 (62)
Sex
Females	129 (30)	16,400 (52)	121 (33)	414 (40)
Males	297 (70)	14,980 (48)	240 (67)	629 (60)
Site of primary cancer, n (%):				
Urogenital	93 (22)		93 (37) *	160 (63) *
Skin	50 (12)		50 (18) *	221 (82) *
Colorectal	39 (9)		39 (19) *	171 (81) *
Lymphomas	53 (12)		53 (38) *	88 (62) *
Respiratory	29 (7)		29 (16) *	149 (84) *
Upper gastrointestinal	25 (6)		25 (19) *	104 (81) *
Breast	45 (11)		45 (61) *	29 (39) *
Cancer of unknown primary	8 (2)		8 (16) *	42 (84) *
Gynaecological	8 (2)		8 (20) *	33 (80) *
Head & neck	5 (1)		5 (19) *	21 (81) *
Neurological	†		†	9 (†)
Endocrine	†		†	†
Bone	†		†	5 (†)
Eye	†		†	†

## Discussion

To our knowledge, this is the first study to quantify the extent to which people with cancer can be ascertained using medicine-dispensing data, controlling for important demographic, data, and calendar time characteristics. We have also detailed how ascertainment of cancer diagnoses using medicine-dispensing records varies depending on cancer site. These findings are particularly relevant for Australian studies using PBS data—that contain no diagnoses information and are often not linked with any other health data holdings. Similar issues are likely to be encountered in other jurisdictions, where strict privacy laws limit the use of data linkage [26].

Previous Australian studies used dispensing of chemotherapy items as recorded in PBS data to identify people with colorectal, lung, and breast cancers and reported sensitivities of 34%, 28%, and 65%, respectively [20, 21]. These estimates are similar to those we report in the present study, though our sensitivity estimates for colorectal and respiratory cancers are lower (22.6% and 23.1%, respectively). These prior studies also reported specificities similar to ours, suggesting that, while many people with a cancer diagnosis are not detected when using the dispensing proxy, those that are identified are very likely to have a cancer diagnosis. Depending on the research question under investigation, this specific feature of the proxy may still be of value beyond its lack of sensitivity. The prior studies concluded that using dispensing records alone to identify people with cancer diagnoses was a poor proxy; and, having expanded the analysis to include a variety of cancer types while adjusting for patient, data, and calendar time characteristics, our findings support the conclusions of the previous research. Our study is also the first to provide estimates of NPV, which further highlight the poor discriminatory power of the proxy.

The poor sensitivity estimates of the dispensing proxy we found may result from the fact that not all cancers are treated with anticancer medicines. Many early-stage cancers are treated with surgical procedures or radiotherapy. Several studies have examined the validity of a range of administrative and registry-based data sources for ascertaining cancer cases in the absence of cancer registry data and found that, broadly, diagnosis codes were needed for more accurate identification of cancer cases [26-33]. Increasing the number of data sources and/or codes used for identification improved the performance of the examined detection algorithms, but these studies have also reported differences in ascertainment by cancer site [28, 29, 34]. Previous Australian studies have shown that hospital admissions data performed best in identifying incident cases of lung and colorectal [20], upper gastrointestinal [35], pancreas [36], and breast cancers [21].

The implications of using this proxy in practice were illustrated in our example opioid initiator cohort. Being able to reliably identify people with cancer is important as they may have unique medicine use patterns and outcomes. In our example, where we sought to identify and exclude people with a cancer diagnosis at opioid initiation, we would have included three quarters of the clients with a cancer diagnosis based on the dispensing proxy with 12-month lookback. With a shorter lookback period, the proportion of clients with cancer misclassified by the proxy increased. The dispensing proxy also identified a disproportionate number of breast cancers (61% correctly identified) but misclassified large proportions of clients with other cancer diagnoses, such as respiratory cancers (84% misclassified). Clients with cancer identified by the proxy were slightly younger than those not identified using the dispensing proxy. Depending on the population being studied, this could lead to biased cohorts and results. In addition, changes in treatment practices over time can have impact on how well dispensing proxies can capture certain indications [2].

Opioids are prescribed to a diverse population, of which people with cancer comprise a potentially small proportion. In our example study, clients with a cancer diagnosed during the 12 months preceding opioid initiation who were misclassified as not having cancer diagnosis accounted for just 3% of non-cancer opioid initiators. Their inclusion would likely have had a negligible impact on findings related to opioid initiators without a recent cancer history. However, clients with a cancer diagnosis at any time prior to opioid initiation who were misclassified as not having a diagnosis comprised 14% of proxy-classified non-cancer opioid initiators. Depending on the aims of a study employing this proxy, the available data, and the relevant time frame for cancer diagnosis, the potential for bias is something researchers must consider when designing their study.

Furthermore, if our intention had been to compare patterns of opioid use and/or outcomes between cancer and non-cancer opioid initiators, or to report on the treatment and outcomes of cancer patients using opioids, our results would likely have been considerably biased, as the proxy would have missed the majority of opioid initiators with cancer. The proxy also identified a larger proportion of patients with breast cancers, which may have created additional biases related to sex. Our DVA sample was majority male and, therefore, biases in population-based samples could be larger. Depending on the cohort under consideration, there is a potentially larger danger in using this proxy to specifically identify and report on people with cancer as opposed to using the proxy to exclude patients. 

### Strengths and limitations

In addition to the sensitivity, specificity and PPV, we were also able to calculate the NPV of the anticancer medicines dispensing proxy. We used matching to ensure that comparisons were made between similar people, during similar time periods, with similar available data from which to ascertain cancer status.

Our study population was comprised of NSW DVA clients aged ≥65 years, which may not represent the entire population of people diagnosed with cancer, especially in relation to cancers that are common in younger people. However, more than two-thirds of new cancers in Australia are diagnosed in people aged ≥60 years [37]. Older patients with cancer are known to receive less chemotherapy and our estimates of sensitivity, specificity, PPV, NPV may be lower than would be observed in a younger cohort [38]. The DVA cohort is eligible for some additional items through the RPBS that are not subsidised under the PBS for the general population; however, there are no additional anticancer medicines on the RPBS that are not subsidised through the PBS. The DVA population may not be representative of people from culturally and linguistically diverse background, though DVA clients have been shown to have similar rates of health service and medicines use to individuals of similar age from the general Australian population [39]. Some cancer sites may be underrepresented in the DVA data, however, the DVA cohort has been shown to have largely similar cancer incidence and mortality rates than the NSW general population [40].

Finally, RPBS data include subsidised medicines dispensed in community pharmacies and medicine use in private hospitals. Inpatient medicine use in public hospitals is not captured. However, most systemic anticancer therapy is administered in the outpatient (non-admitted) setting and is, therefore, captured in RPBS data [22]. Similarly, clients with short follow-up times and those with longer stays in public hospital may have received anticancer medicines that we do not ascertain in RPBS data, meaning our estimates may be slightly lower than the true values.

## Conclusions

The use of anticancer dispensing to identify people with a cancer diagnosis performs poorly as a proxy for cancer status. Depending on the research question and the population under consideration, studies using such a proxy may introduce substantial biases in their findings.

## Disclaimer

The Australian Government Department of Veterans’ Affairs is the copyright owner of the data presented in this manuscript including all tables, figures and supplementary content. The authors have obtained permission from the Australian Government Department of Veterans’ Affairs to publish this manuscript. The views expressed in this version of the work do not necessarily represent the views of the Minister for Veterans’ Affairs or the Department of Veterans’ Affairs. The Commonwealth of Australia does not give any warranty nor accept any liability in relation to the contents of this work.

## Availability of data and materials

Access to the datasets analysed during the current study is not permitted without the express permission of the approving human research ethics committees and data custodians.

## Ethics statement

Our study was approved by the NSW Population and Health Services Research Ethics Committee (Approval Number: 2013/11/494) and The Departments of Defence and Veterans’ Affairs Ethics Committee (Approval number: E013/015). The Ethics Committees granted a waiver of the usual requirement for individual consent to the use of their health information in a research project, in line with the National Statement on Ethical Conduct in Human Research (2007) (Chapter 2.3) and the Guidelines approved under Section 95/95A of the Privacy Act 1988.

## Footnotes

1 For cancers diagnosed longer than 12 months prior to opioid initiation, the median time from diagnosis to opioid initiation was 41.7 months (interquartile range: 25.7 – 61.9)

## Abbreviations

ATC	Anatomic Therapeutic Chemical Classification System
DVA	Australian Government Department of Veterans Affairs
ICD	International Classification of Diseases
NPV	Negative predictive value
NSW	New South Wales
NSWCR	New South Wales Cancer Registry
PBS	Pharmaceutical Benefits Scheme
PPV	Positive predictive value
RPBS	Repatriation Schedule of Pharmaceutical Benefits
